# A clinical-radiomics nomogram for the prediction of the risk of upper gastrointestinal bleeding in patients with decompensated cirrhosis

**DOI:** 10.3389/fmed.2024.1308435

**Published:** 2024-07-31

**Authors:** Zhichun Li, Qian He, Xiao Yang, Tingting Zhu, Xinghui Li, Yan Lei, Wei Tang, Song Peng

**Affiliations:** ^1^Chongqing Health Center for Women and Children, Women and Children’s Hospital of Chongqing Medical University, Chongqing, China; ^2^Sichuan Key Laboratory of Medical Imaging, Department of Radiology, Affiliated Hospital of North Sichuan Medical College, Nanchong, China; ^3^Department of Clinical Laboratory, Affiliated Hospital of North Sichuan Medical College, Nanchong, China

**Keywords:** liver cirrhosis, upper gastrointestinal bleeding, sarcopenia, MDCT, radiomics, nomogram

## Abstract

**Objective:**

To develop a model that integrates radiomics features and clinical factors to predict upper gastrointestinal bleeding (UGIB) in patients with decompensated cirrhosis.

**Methods:**

104 decompensated cirrhosis patients with UGIB and 104 decompensated cirrhosis patients without UGIB were randomized according to a 7:3 ratio into a training cohort (*n* = 145) and a validation cohort (*n* = 63). Radiomics features of the abdominal skeletal muscle area (SMA) were extracted from the cross-sectional image at the largest level of the third lumbar vertebrae (L3) on the abdominal unenhanced multi-detector computer tomography (MDCT) images. Clinical-radiomics nomogram were constructed by combining a radiomics signature (Rad score) with clinical independent risk factors associated with UGIB. Nomogram performance was evaluated in calibration, discrimination, and clinical utility.

**Results:**

The radiomics signature was built using 11 features. Plasma prothrombin time (PT), sarcopenia, and Rad score were independent predictors of the risk of UGIB in patients with decompensated cirrhosis. The clinical-radiomics nomogram performed well in both the training cohort (AUC, 0.902; 95% CI, 0.850–0.954) and the validation cohort (AUC, 0.858; 95% CI, 0.762–0.953) compared with the clinical factor model and the radiomics model and displayed excellent calibration in the training cohort. Decision curve analysis (DCA) demonstrated that the predictive efficacy of the clinical-radiomics nomogram model was superior to that of the clinical and radiomics model.

**Conclusion:**

Clinical-radiomics nomogram that combines clinical factors and radiomics features has demonstrated favorable predictive effects in predicting the occurrence of UGIB in patients with decompensated cirrhosis. This helps in early diagnosis and treatment of the disease, warranting further exploration and research.

## 1 Introduction

Cirrhotic patients often experience malnutrition due to reduced food intake, malabsorption, and decreased protein synthesis, leading to a decrease in both the quantity and quality of skeletal muscle (1, 2). Sarcopenia is a condition that results in decreased muscle mass and quality, and it is a common complication of cirrhosis (3). Cirrhotic patients with sarcopenia are at a higher risk of experiencing reduced quality of life, associated complications, and lower survival rates compared to cirrhotic patients without sarcopenia (3, 4). Sarcopenia can be used as a predictor of the occurrence and prognosis of complications in cirrhosis (4, 5).

As we know, patients with decompensated cirrhosis usually have clinical symptoms such as portal hypertension, ascites, esophagogastric fundal vein varices (GEVs), and splenomegaly. The presence of cirrhosis and ascites can increase the likelihood of intra-abdominal hypertension (IAH) (6). Elevated intra-abdominal pressure can cause abdominal muscle spasms, fatigue, and decreased anti-tension. Prolonged abdominal hypertension can cause degeneration and atrophy of the abdominal muscles. In patients with cirrhotic portal hypertension, elevated intra-abdominal pressure (IAP) may have deleterious effects on oesophageal variceal hemodynamics, significantly increasing variceal pressure and wall tension (7). In addition, decompensated cirrhotic patients with concomitant IAH and abdominal muscle weakness are at higher risk of developing umbilical or abdominal wall hernias (8). Based on the above reports, it seems that changes in abdominal muscles could be linked to the development and advancement of complications related to cirrhosis. Additionally, measuring the skeletal muscle at the L3 level may serve as an indicator of the patient’s overall body mass and nutritional status in individuals with cirrhosis.

About 50% of cirrhotic patients have gastroesophageal varices (GEV), and 25–35% of those patients will experience upper gastrointestinal bleeding (UGIB) (9, 10). Liver cirrhosis complicated by UGIB is a dangerous and rapidly progressing condition that can cause severe bleeding, shock, and acute peripheral circulatory failure, with a high lethality rate (11). The gold standard for clinical assessment of UGIB and GEV is endoscopy (12). However, due to the endoscopy’s invasive nature, some cirrhotic patients cannot tolerate this procedure because it may induce varicose vein bleeding (13). Therefore, simpler, non-invasive alternatives to endoscopy are needed to predict the risk of UGIB in patients with decompensated cirrhosis.

Current non-invasive methods for predicting UGIB include serological markers, imaging indicators, elastography and combinations of various indicators. Serological markers, such as von willebrand factor (vWF), vitro score, platelet count (PLT), and prothrombin time (PT), lack the desired sensitivity and specificity, requiring further validation for practice use (14–16). In addition, the measurement of the portal vein, splenic vein diameter, blood flow velocity, blood flow volume, and other abdominal ultrasound indicators for predicting oesophageal varices and bleeding risk can be easily influenced by the operator’s own experience and subjectivity (17, 18). Transient elastography (TE) indirectly reflects the hepatic venous pressure gradient (HVPG) value by measuring liver stiffness to predict the degree of portal hypertension and the risk of bleeding (19), but the interference of ascites, obesity, gastrointestinal gas, and other factors may lead to errors in prediction.

MDCT is one of the routine investigations for cirrhosis, aiding in the diagnosis and evaluation of cirrhosis complications. Previous studies have analyzed quantitative CT indicators and radiomics of the liver, spleen, and esophagogastric fundal veins to predict UGIB risk in cirrhotic patients (20, 21). As far as I know, no studies have been conducted to investigate the relationship between quantitative CT indicators, radiomics of abdominal muscles, and the risk of UGIB in decompensated cirrhotic patients. Hence, the study aimed to create a non-invasive method based on MDCT to predict UGIB in decompensated cirrhotic patients by integrating clinical factors and radiomics features of abdominal muscles.

## 2 Materials and methods

### 2.1 Patients and data acquisition

**Figure 1:**
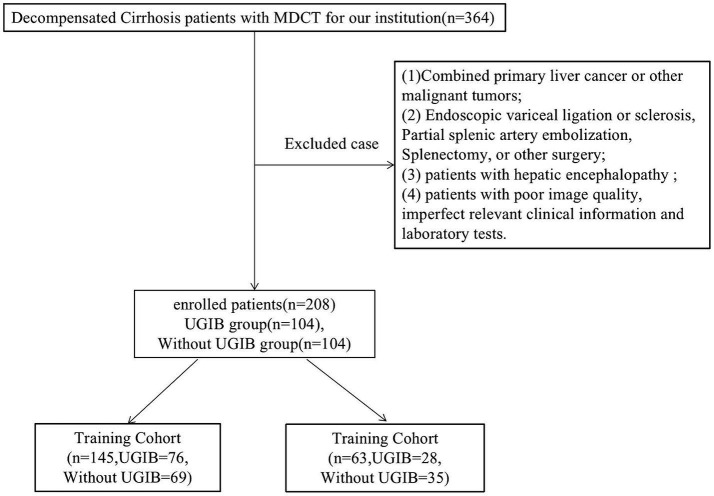
Flowchart of inclusion and exclusion procedures for this study.

The hospital ethics review board committee approved this retrospective study [Number 2022ER322-1], and the informed consent was waived. This study screened 208 patients with decompensated cirrhosis in our hospital from January 2019 to May 2023, based on inclusion and exclusion criteria. Criteria for inclusion: (1) age ≥ 18 years; (2) patients with decompensated cirrhosis, diagnostic criteria based on 2019 revised diagnostic and treatment guidelines for liver cirrhosis of the Chinese Medical Association, hepatology branch (22); (3) patients did not have upper gastrointestinal bleeding within 1 year prior to admission; (4) patients received endoscopy and whole abdomen MDCT scan after admission, and completion of a CT scan within a week before endoscopy; and (5) the relevant clinical information and laboratory tests were complete. Criteria for exclusion: (1) combined primary liver cancer or other malignant tumors; (2) patients have previously undergone any of the following: endoscopic variceal ligation or sclerosis, partial splenic artery embolization, splenectomy, or other surgery; (3) patients with hepatic encephalopathy; and (4) patients with poor image quality, imperfect relevant clinical information, and laboratory tests. See Figure 1 for the patient selection flowchart.

### 2.2 Patient clinical information

By reviewing the medical records of all patients, these clinical data including age, sex, body mass index (BMI), and routine blood and coagulation indexes within 24 h after admission, such as PLT, hemoglobin, total bilirubin, albumin, PT, international normalized ratio (INR), creatinine, alanine aminotransferase (ALT) and aspartate aminotransferase (AST), were reviewed and collected in medical records. Based on clinical signs and imaging examination results, ascites were graded into 3 grades according to the European Association for the Study of the Liver (EASL) criteria (23), grade 1: a small amount of ascites that can only be detected by ultrasonography or MDCT; grade 2: moderate amount of ascites with moderate and symmetrical abdominal distention; and grade 3: a large amount of ascites with significant abdominal distention. Liver function is graded based on the Child-Pugh score, which takes into account clinical features such as ascites and encephalopathy, as well as laboratory values such as serum albumin, bilirubin, and PLT (24).

### 2.3 Endoscopy

Endoscopy is used as the gold standard for UGIB and ruling out bleeding caused by ulcers. The criteria for bleeding in this study were based on one or more of the following signs: (1) active oozing or bleeding at the site of the varices, with no other bleeding sources during endoscope; and (2) finding a thrombus head on a visible variceal vein without finding a bleeding lesion elsewhere (12). Endoscopy was performed by an experienced gastroenterologist. All the patients who underwent endoscopy varices were classified as small varices<5 mm, and large varices>5 mm. Red color sign (RC) was defined that the surface of oesophageal varices showed red wale marking (RWM), cherry red spot (CRS), hematocystic spot(HS), and diffuse redness(DR) on endoscopy.

### 2.4 CT scan parameters and image acquisition

The scan was performed using a GE LightSpeed VCT 64-slice spiral CT, with the range from the diaphragm to the pubic symphysis. The scanning parameters are as follows: tube voltage of 120 Kv, tube current ranging from 250 to 300 mA, scan time of 0.5 s/360°, pitch of 1.0, acquisition slice thickness of 1.0 mm, reconstructed slice thickness of 5 mm, matrix size of 512 × 512. A nonionic iodinated contrast medium with an iodine concentration of 370 mg/mL (Yangzijiang, Jiangsu, China) was intravenously injected at a rate of 4 mL/s using a power injector with a dose of 2 mL/kg body weight, with an upper limit of 100 mL per patient. An abdominal pre-contrast CT scan was performed first, followed by two post-contrast CT scans during the arterial phase (25–30S) and the venous phase (45–50S).

On CT images, we measured the spleen diameter (SD) and spleen thickness (ST). The SD was defined as the longest diameter of the spleen at the central level of the splenic hilum (anteroposterior straight line). The ST was defined as the shortest diameter from the inner margin to the outer margin of the spleen at the central level of the splenic hilum.

### 2.5 The measurement of the abdominal skeletal muscle area and density, and subcutaneous fat area

**Figure 2:**
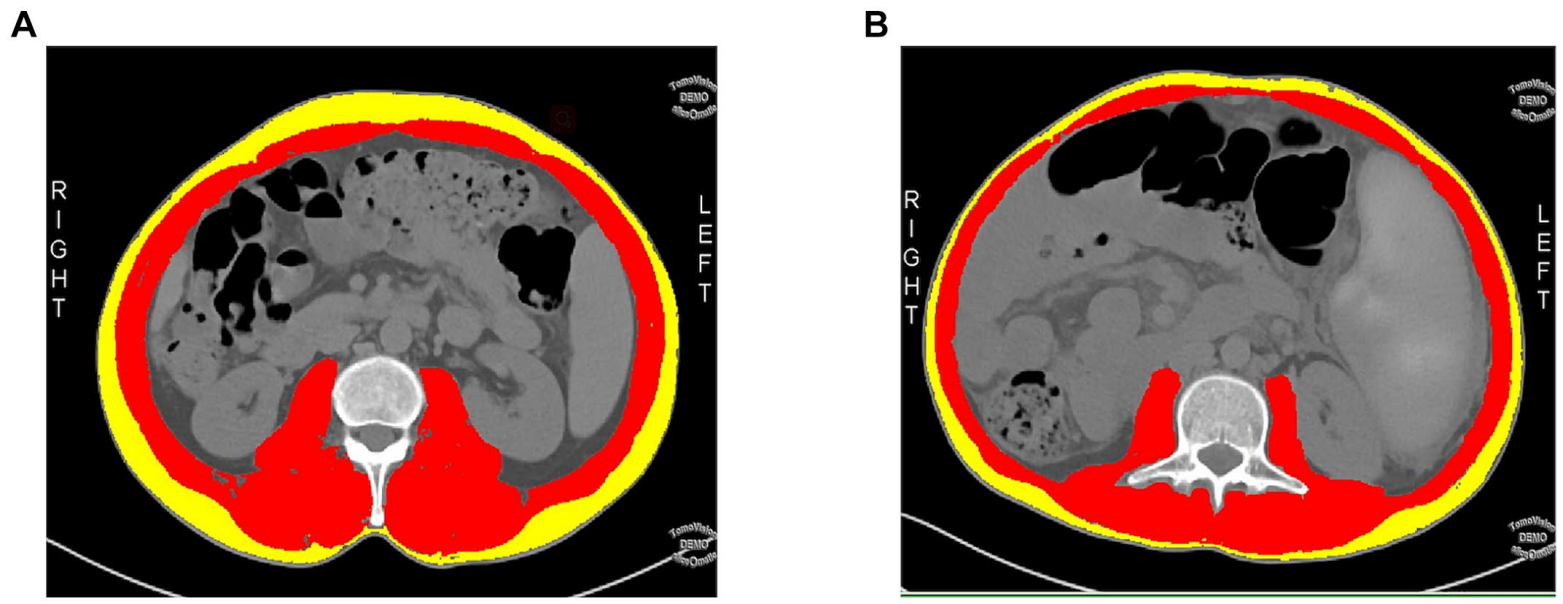
A cross-sectional abdominal skeletal muscle area (SMA, cm^2^; red areas), surrounding subcutaneous fat area (SFA; yellow areas) and skeletal muscle density (SMD, HU) were measured on abdominal cross-sectional MDCT images at the maximum level of the third lumbar vertebra(L3) using sliceomatic software. **(A)** A 48-year-old male patient with a decompensated cirrhosis without upper gastrointestinal bleeding(UGIB), SMA = 148.9 cm^2^, SFA = 68.09 cm^2^, SMD = 47.99HU. **(B)** A 49-year-old male patient with decompensated cirrhotic patient with UGIB, SMA = 115 cm^2^, SFA = 38.28 cm^2^, SMD = 41.53HU.

The Slice-O-Matic software (5.2.1 version, https://tomovision.com/) was used to semi-automatically identify the entire abdominal wall muscles at the maximum level of the L3 according to the threshold of −29 to 150 HU, and the subcutaneous fat according to the threshold of-30 to-190 HU, respectively. After determining the internal and external contours of the muscles, they were separated from the subcutaneous fat and abdominal fat. Any misidentified areas in the images were corrected by manual adjustment. The subcutaneous fat area (SFA, cm^2^), skeletal muscle area (SMA,cm^2^), and skeletal muscle density (SMD, HU) of the abdomen were automatically calculated and obtained (25). For details, see Figure 2. This work was completed by radiologists (reader1and reader2) with experiences in diagnostic abdominal imaging, and the average of their two measurements was taken. The endoscopy results were not shared with the two radiologists.

The skeletal muscle index (SMI) is used for the evaluation of sarcopenia. The SMI was calculated using the formula SMI=SMA (cm^2^)/height^2^ (m^2^). We used the diagnostic criteria for sarcopenia in the Chinese population based on SMI, as reported by Zeng et al. (26), men with an SMI of less than 44.77 cm^2^/m^2^ and women with an SMI of less than 32.50 cm^2^/m^2^ were defined as sarcopenia.

### 2.6 Feature extraction and selection of radiomics

The cross-section of abdominal muscles at the maximal level of the L3 was selected as the regions of interest (ROI). ROI was outlined semi-automatically using 3D slicer software (4.11.2 version, https://www.slicer.org/), and manually adjusted to guarantee utmost accuracy. Using R software (4.2.2 version, http://www.R-project.org), we extracted radiomics features from each ROI. For each patient’s ROI, 1223 radiomics features describing the L3-skeletal muscle’s internal and surface texture were extracted.

To analyze inter-and intra-observer reliability, 68 cases were selected randomly from the total sample. After 1 month, two radiologists segmented and extracted features from these images separately. An intraclass correlation coefficient(ICC) value above 0.75 was considered highly reproducible for reliability assessment, features with ICC values exceeded 0.75 were included in subsequent analysis (27).

The large number of redundant features obtained after feature extraction leads to overfitting and reduces the discriminative power of the model. LASSO regression allows active selection on large sets of multicollinear variables and uses collapsed cross-validation to select the most effective predictive features (28). Final filtered features were weighted using the LASSO algorithm to generate a Rad-score by linear combination, and then a radiomics model was built.

### 2.7 Radiomics nomogram construction

The Rad-scores and the clinical variables (clinical data and CT quantified features) were tested in univariate logistic regression analysis. All variables with *p* < 0.05 were entered into the multivariate logistic regression analysis. A radiomics nomogram was then constructed according to the multivariate logistic regression model.

### 2.8 Model performance assessment

Plotting and calculating receiver operating characteristic curves (ROC), accuracy, sensitivity, specificity, positive predictive value (PPV), negative predictive value (NPV), and F1-score for each model to assess the generalization ability of the model. The area under the ROC curve (AUC) of the three models was compared using the Delong test. If the *p*-value is less than 0.05, it is considered to have statistical significance. Then, the nomogram was evaluated using calibration curves. Decision curve analysis (DCA) can assess the usability of a model and show the “net benefit” of a model (29). Therefore, we used DCA to analyze and compare the performance of the three models in terms of clinical utility.

### 2.9 Statistical analysis

The clinical data was analyzed using SPSS (version 27.0, IBM, Armonk, NY). Radiomics feature and model performance were analyzed using R. Continuous variables are expressed as mean ± standard deviation (SD), while categorical variables are expressed as numbers and percentages. Normal distribution of continuous parameters was compared using independent samples *t*-test, and non-normal distribution was compared using Mann–Whitney U test. Categorical parameters were compared using chi-squared test. Univariate analyses were conducted to identify risk factors associated with the development of UGIB in patients with decompensated cirrhosis. Significant indicators from the univariate analyses were included in multivariate logistic regression analyses. Results with *p* < 0.05 were considered statistically significant.

LASSO regression analyses of radiomics features were performed using the R package “glmnet.” Nomograms and calibration curves were generated using the “rms” software package. Finally, the “dca.r” package is used to calculate the DCA.

## 3 Results

### 3.1 Clinical factors selection and construction of the clinical model

ICC results showed excellent consistency (all ICC > 0.75, *p* < 0.001) between radiologist 1 and radiologist 2 for intra-and interobserver measurements of SMA, SFA, and SMD values on MDCT (Table 1).

**Table 1:** Consistency within and between observers in terms of SMA and SFA.

Variables	ICCs	95%CI		*p*	ICCs	95%CI		*p*
		Lower limit	Upper limit			Lower limit	Upper limit	
SMA (cm^2^)	0.984	0.977	0.988	<0.001	0.990	0.987	0.992	<0.001
SFA (cm^2^)	0.916	0.890	0.936	<0.001	0.934	0.913	0.950	<0.001
SMD (HU)	0.945	0.926	0.959	<0.001	0.959	0.943	0.970	<0.001

SMA, skeletal muscle area; SFA, subcutaneous fat area; SMD, skeletal muscle density; ICCs, inter-and intra-group correlation coefficients; CI, confidence interval.

Table 2 showed the fundamental characteristics of the patients in the training (*n* = 145) and validation (*n* = 63) cohort. Univariate logistic regression showed that the Child-Pugh score grade, Albumin, PT, INR, PLT, AST, SD, SMA, SMD, and sarcopenia between decompensated cirrhotic patients with and without UGIB were significant differences in the training cohorts (*p* < 0.05), multivariate logistic regression analysis showed that sarcopenia and PT could be used as independent risk factors to predict UGIB in patients with decompensated cirrhosis (*p* < 0.05) (Figure 2; Table 3). The AUC for clinical factors was 0.822 (95% *CI* 0.753–0.891) in the training cohort and 0.756 (95% CI 0.634–0.877) in the validation cohort (Table 4).

**Table 2:** Baseline characteristics of decompensated cirrhotic patients with and without UGIB in the training and validation cohorts.

Variables	Training Cohort (*n* = 145)		*p*	Validation Cohort (*n* = 63)		*p*
	UGIB (*n* = 76)	Without UGIB (*n* = 69)		UGIB (*n* = 28)	Without UGIB (*n* = 35)	
Age(y), mean ± SD	57.3 ± 12.27	55.59 ± 10.83	0.377	57.61 ± 12.84	56.2 ± 10.19	0.629
Sex, male, *n* (%)	50 (65.8)	39 (56.5)	0.252	13 (46.4)	29 (82.9)	0.002
BMI (kg/m2)	22.49 (20.79, 25.08)	23.44 (21.07, 25.16)	0.402	22.33 (20.35, 25.76)	23.44 (21.16, 25.34)	0.59
Total Bilirubin (μmol/L)	28.35 (17.83, 50.5)	33 (17.95, 47.85)	0.954	24.05 (14.85, 40.93)	20.1 (13.9, 50.6)	0.879
Albumin(g/L)	30.44 ± 4.31	33.85 ± 5.74	<0.001	29.1 ± 4.53	34.34 ± 6.09	<0.001
ALT(U/L)	27.5 (18.3, 53.9)	35 (23, 53)	0.17	20.5 (13, 27.5)	27 (20, 55)	0.015
AST (U/L)	40.5 (26, 67)	52 (35.5, 91)	0.03	30 (20.5, 43.25)	34 (23, 72)	0.171
Creatinine (μmol/L)	69.85 (56.75, 79.48)	59.8 (51.4, 73.8)	0.016	60.3 (47.15, 75.85)	63 (51.5, 74.8)	0.52
PT(s)	18.05 (16.9, 19.4)	15.60 (14.2, 17.6)	<0.001	18 (16.68, 18.9)	15.4 (13, 17.5)	<0.001
INR (%)	1.45 (1.29, 1.67)	1.36 (1.2, 1.54)	0.005	1.43 (1.36, 1.59)	1.36 (1.2, 1.49)	0.062
Hemoglobin (g/L)	81.5 (65, 100)	108 (86, 121)	<0.001	71.5 (58.25, 84)	111 (80, 128)	<0.001
PLT (10^9^/L)	72 (60.25, 83)	83 (71, 98.5)	<0.001	75.5 (57.25, 83.75)	88 (79, 100)	0.01
Child-Pugh *n* (%)			0.001			0.001
A	5 (6.6)	19 (27.5)		0	14 (40)	
B	42 (55.3)	38 (55.1)		18 (64.3)	14 (40)	
C	29 (38.2)	12 (17.4)		10 (35.7)	7 (20)	
Endoscopy						
Large varices (*n*,%)	62 (81.6%)	40 (58%)	0.002	23 (82.1)	19 (54.3)	0.031
Small varices (*n*,%)	14 (18.4%)	29 (42%)		5 (17.9)	16 (45.7)	
Red (+)	61 (80.3)	24 (34.8)	<0.001	23 (82.1)	15 (39.5)	0.002
**MDCT**						
SMA (cm^2^)	106.08 ± 20.64	118.7 ± 26.4	0.002	96.92 ± 17.97	122.79 ± 22.62	<0.001
SFA (cm^2^)	73.99 (39.36, 132.35)	84 (43.56, 156.85)	0.346	78.53 (33.04, 133.2)	73.91 (34.66, 114.5)	0.825
SMD(HU)	36.27 ± 7.96	40 ± 5.8	0.002	34.67 ± 8.28	40.36 ± 6.79	0.004
SMI	39.51 (35.59, 44.05)	44.12 (38.92, 52.83)	<0.001	38.27 (34.33, 42.89)	44.92 (40.46, 49.22)	<0.001
Sarcopenia, *n* (%)	47 (61.8)	15 (21.7)	<0.001	17 (60.7)	11 (31.4)	0.02
SD (mm)	15.14 ± 2.12	14.3 ± 2.72	0.047	15.96 ± 2.26	14.43 ± 2.22	0.09
ST (mm)	5.62 ± 1.23	5.19 ± 1.22	0.036	5.92 ± 1.05	5.33 ± 1.13	0.035

UGIB, upper gastrointestinal bleeding; BMI, body mass index; ALT, alanine aminotransferase; AST, aspartate aminotransferase; PT, prothrombin time; INR, international normalized ratio; PLT, platelet count; SMA, skeletal muscle area; SFA, subcutaneous fat area; SMD, skeletal muscle density; SMI, skeletal muscle index; SD, spleen diameter; ST, spleen thickness.

**Table 3:** Univariate and multivariate logistic regression analysis of patients with decompensated cirrhosis combine with UGIB in the training cohorts.

	Univariate logistic regression		Multivariate logistic regression	
	OR (95%CI)	*p*	OR (95%CI)	*p*
Albumin(g/L)	0.869 (0.806–0.937)	<0.001		
PT(s)	1.379 (1.187–1.602)	<0.001	1.538 (1.198–1.974)	0.001
INR (%)	4.950 (1.501–16.331)	0.009		
PLT	0.971 (0.954–0.988)	0.001		
Child-Pugh, *n* (%)	1.439 (1.197–1.730)	<0.001		
B	4.2 (1.429–12.348)	0.009		
C	9.183 (2.786–30.275)	<0.001		
SMA (cm^2^)	0.977 (0.963–0.992)	0.002		
SMD(HU)	0.925 (0.88–0.973)	0.003		
Sarcopenia, *n* (%)	5.834 (2.795–12.178)	<0.001	5.555 (2.037–15.147)	0.001
SD (mm)	1.338 (1.016–1.763)	0.038		
Rad score	2.718 (1.929–3.828)	<0.001	3.024 (1.953–4.681)	<0.001

UGIB, upper gastrointestinal bleeding; PT, prothrombin time; PLT, platelet count; SMA, skeletal muscle area; SMD, skeletal muscle density; SD, spleen diameter; OR, odds ratio.

**Table 4:** The AUC of radiomics model, clinical model, and nomogram model for predicting UGIB of the decompensated cirrhosis patients in the training and validation cohorts.

	AUC (95%CI)	Accuracy, %	Sensitivity, %	Specificity, %	PPV, %	NPV, %	F1, %
Training cohort							
Radiomics model	0.830 (0.762–0.897)	77.9	80.3	75.4	78.2	77.6	79.2
Clinical model	0.822 (0.753–0.891)	74.5	73.7	75.4	76.7	72.2	75.2
Nomogram model	0.902 (0.850–0.954)	82.1	85.5	78.3	81.3	83.1	83.3
Validation cohort							
Radiomics model	0.765 (0.644–0.886)	68.3	81.8	53.3	65.9	72.7	73
Clinical model	0.756 (0.634–0.877)	66.7	60.6	73.3	71.4	62.9	65.6
Nomogram model	0.858 (0.762–0.953)	79.4	75.8	83.3	83.3	75.8	79.4

AUC, the area under the curve; PPV, positive predictive value; NPV, negative predictive value.

### 3.2 Radiomics model establishment

**Figure 3:**
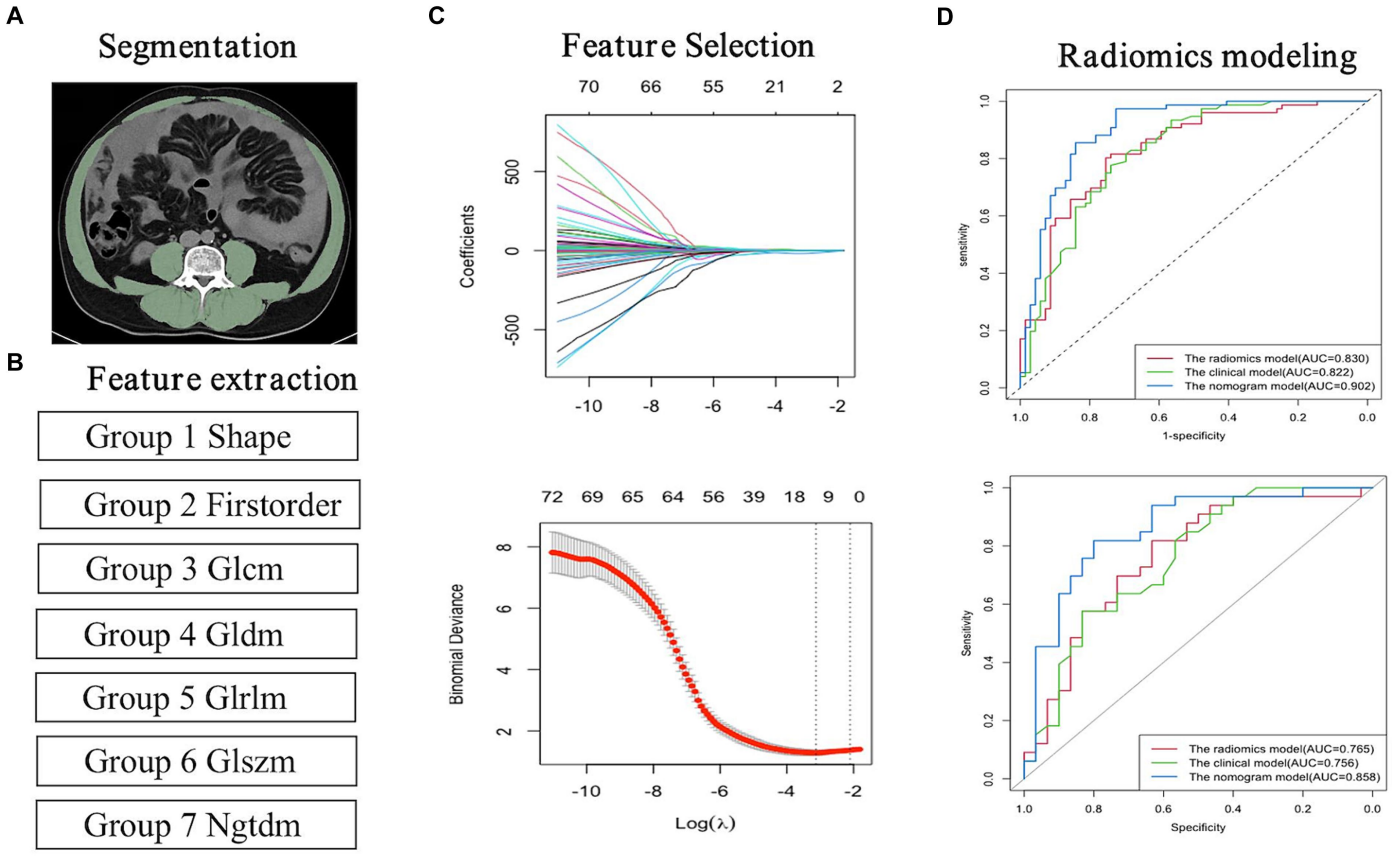
Flowchart for the radiomics analysis. **(A)** Image segmentation; **(B)** Feature extraction; **(C)** Feature selection and model building. **(D)** Radiomics model building.

Of the 1,223 radiomics features extracted, 1,082 features were proved to have good inter-and intra-observer agreement with ICCs >0.75. 88 radiomics features showing significant differences between decompensated cirrhotic patients with and without UGIB (*p* < 0.05) were incorporated into the LASSO logistic regression to determine the best valuable features (Figure 3). Eventually, 11 different features were screened to form the radiomics signature (Figure 3; Table 5). Radiomics features showed good predictive accuracy, with an AUC of 0.830 (95% *CI*, 0.762–0.897) in the training cohort and 0.765 (95% *CI*, 0.644–0.886) in the validation cohort (Table 4).

**Table 5:** Information on radiomics features after dimensionality reduction and feature selection by LASSO regression.

3rd lumbar vertebra level muscles on MDCT			
NO.	Radiomics features	NO.	Radiomics features
N5	Shap (Maximum2DDiameterColumn)	N680	Firstorder (Skewness)
N83	Glrlm (RunVariance)	N932	Glszm (SizeZoneNonUniformity)
N193	Glszm (ZoneEntropy)	N1009	Glrlm (RunEntropy)
N366	Glszm (GrayLevelNonUniformity)	N1106	Glrlm (RunVariance)
N494	Firstorder (Skewness)	N1123	Glszm (ZoneEntropy)
N671	Firstorder (Kurtosis)		

LASSO, least absolute shrinkage and selection operator; MDCT, multi-detector computer tomography.

### 3.3 The clinical-radiomics nomogram model building and assessment of the performance of different model

**Figure 4:**
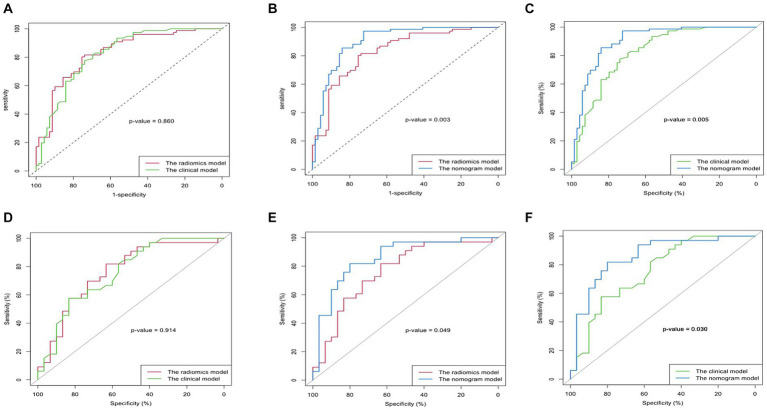
Comparison of the AUC of the clinical model, radiomics model, and nomogram model in predicting upper gastrointestinal bleeding (UGIB) of the decompensated cirrhosis patients [**(A–C)** Training set; **(D–F)** Validation set]. The nomogram model had better diagnostic performance than the radiomics model (*p* < 0.05) and the clinical model (*p* < 0.05), but the radiomics signature’s diagnostic performance did not differ significantly from that of the clinical factors (*p* > 0.05) in both training and validation cohort.

The diagnostic performances of the three models were showed in Table 4. The ROC curves of the three models were displayed in Figure 4. It was found that the clinical-radiomics nomogram model demonstrated superior diagnostic performance than the radiomics features (AUC: 0.902 vs. 0.830, *p* = 0.003) and the clinical factors (AUC: 0.902 vs. 0.822, *p* = 0.005), but the radiomics signature’s diagnostic performance was not different significantly from the clinical factors (AUC: 0.830 vs. 0.822, *p* = 0.86), in the prediction of UGIB in decompensated cirrhotic patients (Figure 4).

**Figure 5:**
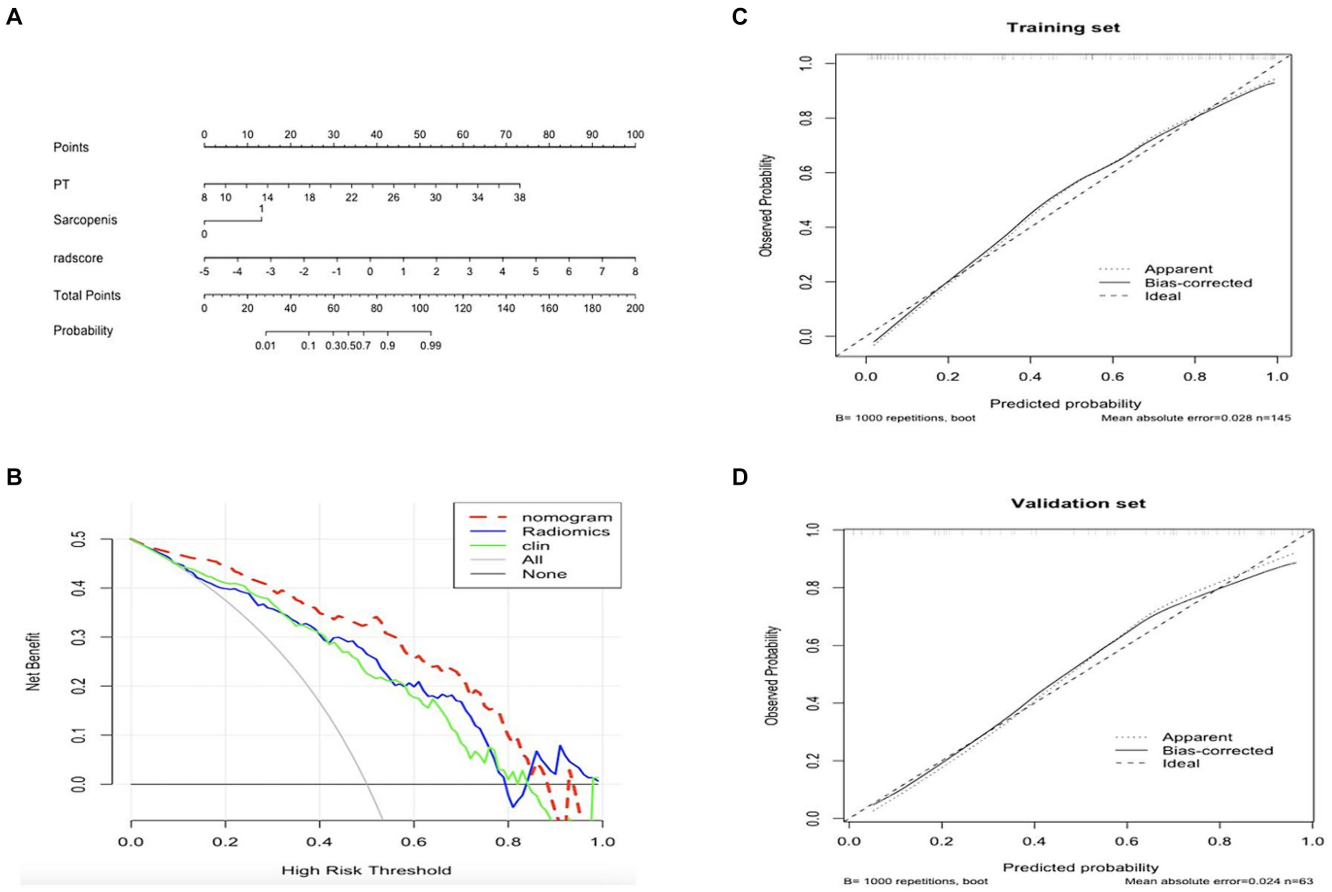
Plotting nomograms, clinical decision curves, and calibration curves to assess the predictive performance of the model. **(A)** The developed nomogram is based on the clinical-radiomics prediction model to predict the risk of upper gastrointestinal bleeding(UGIB) in patients with decompensated cirrhosis. Sarcopenia:1, non-sarcopenia:0. **(B)** Decision curve analysis of the three models in the training group. The light grey line assumes that all patients have the possibility of concomitant UGIB. The black horizontal line assumes that no patients have UGIB. X-axis represents the threshold probability. Y-axis measures the net gain. The blue line represents the radiological model. The green line represents the clinical model. Red line represents the combined model. **(C,D)** Calibration curves for the nomogram in the training and validation cohorts. The curve indicates that the net benefit of the nomogram is better than the other models when the threshold is within the range of 0.05–0.85 in the training cohort. The closer the ideal line fit to the apparent line, the greater the prediction accuracy of the nomogram.

Calibration curves showed better calibration in the training cohort and validation cohort (Figure 5). Results from the DCA suggest that the clinical-radiomics nomogram model provides a greater net benefit for clinical decision-making than the radiomics and clinical models in the training cohort (Figure 5).

## 4 Discussion

Our study constructed and verified a clinical-radiomics nomogram model to predict UGIB in patients with decompensated cirrhosis based on MDCT images of the L3 skeletal muscles. The clinical-radiomics nomogram, which combines radiomics features and clinical factors, demonstrated excellent diagnostic performance in predicting UGIB of decompensated cirrhotic patients.

Sarcopenia is prevalent in patients with liver cirrhosis. Patients with decompensated cirrhosis exhibit a notably elevated occurrence of sarcopenia compared to those with compensated cirrhosis (3). Sarcopenia is strongly associated with complications of liver cirrhosis, such as ascites, oesophageal varices, and hepatic encephalopathy (3–5, 26). However, few studies have reported the correlation between sarcopenia and upper gastrointestinal bleeding in cirrhosis. Topan et al. (30) and Aldo et al. (31) have shown that cirrhotic patients with sarcopenia have a higher incidence of oesophageal varices and variceal bleeding compared to those without sarcopenia. A study by Zeng et al. (26) showed that patients with cirrhosis combined with sarcopenia had a higher incidence of UGIB during the 2-year follow-up period compared to patients without sarcopenia. The results of the above studies are consistent with the results of our study. In our study, sarcopenia was strongly associated with UGIB. Decompensated cirrhotic patients with combined sarcopenia have a 5–6 times higher risk of UGIB compared to those without sarcopenia.

The abdominal SMD and SMA at the maximum level of L3 on MDCT can indirectly reflect the skeletal muscle mass of the whole body, reduced SMD and SMA imply an increased proportion of intermuscular fat and muscular atrophy. EbadiM’s study showed that a reduced abdominal SMD at the maximum level of L3 is negatively correlated with clinical outcomes and strongly associated with complications of portal hypertension in cirrhotic patients (32). Our study found that the abdominal SMA and SMD, although markedly lower in decompensated cirrhotic patients with UGIB than those without UGIB, could not be used as independent hazard factors for forecasting UGIB in patients with decompensated cirrhosis. The reasons may be as follows: due to malnutrition and metabolic factors, the abdominal skeletal muscle in patients with cirrhosis develops fat deposition and atrophy at the same time, and single abdominal SMD and SMA cannot fully represent the pathological changes of skeletal muscle.

Liver cirrhosis in the decompensated phase is primarily characterized by long-term liver function damage, leading to decreased hepatic synthetic capacity and coagulation dysfunction (33–35). Our study found that some laboratory indicators reflecting liver reserve function status were associated with UGIB in patients with decompensated cirrhosis. This conforms to the pattern of disease progression, the more severe the liver function damage, the higher the risk of UGIB. Among these laboratory indicators, only PT could be an independent risk factor for predicting the risk of UGIB in patients with decompensated cirrhosis. The reason may be that PT is the most sensitive and widely used screening test for external coagulation.

With the progression of liver cirrhosis, the spleen becomes enlarged due to tissue hyperplasia, fibrosis, and portal congestion. The size of the spleen may be related to UGIB in patients with liver cirrhosis. In our study, the SD and ST showed a significant difference between patients with and without UGIB in the training cohort. The ST showed a significant difference, and the SD did not show a significant difference between patients with and without UGIB in the validation cohort. The univariate and multivariate analysis results of the training cohort showed that only ST was a risk factor for UGIB, however, it cannot be used as an independent risk factor for UGIB in patients with decompensated cirrhosis. The reason may be that individuals can have variations in the shape, size, and function of their spleen.

Radiomics is a non-invasive image analysis technique that employs high flux throughput feature extraction algorithms to quantitatively assess the distribution characteristics of grey levels and pixels in CT images, thereby revealing differences between individuals that cannot be identified by the human eye (36). We chose MDCT images of skeletal muscles at the level of the L3 vertebrae for feature extraction and combined the radiomics features with clinical features to establish a clinical-radiomics nomogram model. Radiomics can identify muscle features and tissue heterogeneity that are difficult to access through visual assessment, aiding in the early detection of muscle loss and mass loss during disease progression (37). In our study, the nomogram model showed better diagnostic and predictive ability than radiomics models and clinical models alone. The incorporation of both radiomics and clinical data may contribute to the excellent performance of the nomogram model. Such an approach allows for direct consideration of disease status and provides a greater advantage than the traditional MDCT image based on the SMA and SMD to assess the muscle features, resulting in superior model performance. In addition, DCA also validated that a nomogram model based on skeletal muscle mass analysis had more net benefit than clinical and radiomics models in predicting UGIB in decompensated cirrhosis.

There are several shortcomings in our study. First, we used a single-center, small-sample for our study, which may have been a source of bias. Therefore, future studies should consider multi-center studies with a larger sample to ensure more accurate results. Second, the muscle imaging histology model in this study was built based on 2D images, not 3D images. However, the imaging histology prediction model based on 2D images has shown some promising results. Additionally, the time spent on feature extraction and prediction model building was relatively short, making it more convenient and faster than the imaging histology processing of 3D images. Last, diagnostic accuracy in the training cohort in our study is usually overestimated. Therefore, prospective external validation is needed in future studies.

To sum up, the clinical-radiomics model was found to be more accurate in predicting the occurrence of UGIB in decompensated cirrhosis compared to individual clinical or radiomics models. This is valuable for the diagnosis of UGIB in patients with decompensated cirrhosis and could potentially complement the gold standard of endoscopy. Additionally, it offers new insights for evaluating the risk of UGIB in decompensated cirrhotic patients.

## Data availability statement

The original contributions presented in the study are included in the article/supplementary material, further inquiries can be directed to the corresponding authors.

## Ethics statement

The studies involving humans were approved by Medical Ethics Committee of Affiliated Hospital of North Sichuan Medical College. The studies were conducted in accordance with the local legislation and institutional requirements. Written informed consent for participation was not required from the participants or the participants’ legal guardians/next of kin in accordance with the national legislation and institutional requirements.

## Author contributions

ZL: Writing – original draft. QH: Writing – original draft. XY: Writing – review & editing. TZ: Writing – review & editing. XL: Writing – review & editing. YL: Writing – review & editing. WT: Writing – original draft, Writing – review & editing. SP: Writing – review & editing.

## References

[1] Cruz-Jentoft AJ Baeyens JP Bauer JM Boirie Y Cederholm T Landi F. Sarcopenia: European consensus on definition and diagnosis: report of the European working group on sarcopenia in older people. Age Ageing. (2010) 39:412–23. doi: 10.1093/ageing/afq034, PMID: 20392703 PMC2886201

[2] Toshikuni N Arisawa T Tsutsumi M. Nutrition and exercise in the management of liver cirrhosis. World J Gastroenterol. (2014) 20:7286–97. doi: 10.3748/wjg.v20.i23.7286, PMID: 24966599 PMC4064074

[3] Tandon P Montano-Loza AJ Lai JC Dasarathy S Merli M. Sarcopenia and frailty in decompensated cirrhosis. J Hepatol. (2021) 75:S147–62. doi: 10.1016/j.jhep.2021.01.025 34039486 PMC9125684

[4] Kim G Kang SH Kim MY Baik SK. Prognostic value of sarcopenia in patients with liver cirrhosis: a systematic review and meta-analysis. PLoS One. (2017) 12:e0186990. doi: 10.1371/journal.pone.0186990, PMID: 29065187 PMC5655454

[5] Bunchorntavakul C. Sarcopenia and frailty in cirrhosis: assessment and management. Med Clin North Am. (2023) 107:589–604. doi: 10.1016/j.mcna.2022.12.007 37001955

[6] Pereira R Buglevski M Perdigoto R Marcelino P Saliba F Blot S. Intra-abdominal hypertension and abdominal compartment syndrome in the critically ill liver cirrhotic patient-prevalence and clinical outcomes. A multicentric retrospective cohort study in intensive care. PLoS One. (2021) 16:e0251498. doi: 10.1371/journal.pone.0251498, PMID: 33984016 PMC8118291

[7] Escorsell A Gines A Llach J Garcia-Pagan JC Bordas JM Bosch J. Increasing intra-abdominal pressure increases pressure, volume, and wall tension in esophageal varices. Hepatology. (2002) 36:936–40. doi: 10.1053/jhep.2002.35817, PMID: 12297841

[8] Belghiti J Durand F. Abdominal wall hernias in the setting of cirrhosis. Semin Liver Dis. (1997) 17:219–26. doi: 10.1055/s-2007-1007199 9308126

[9] Lesmana CRA Raharjo M Gani RA. Managing liver cirrhotic complications: overview of esophageal and gastric varices. Clin Mol Hepatol. (2020) 26:444–60. doi: 10.3350/cmh.2020.0022, PMID: 33053928 PMC7641566

[10] Jakab SS Garcia-Tsao G. Evaluation and Management of Esophageal and Gastric Varices in patients with cirrhosis. Clin Liver Dis. (2020) 24:335–50. doi: 10.1016/j.cld.2020.04.011, PMID: 32620275 PMC11090175

[11] Feinman M Haut ER. Upper gastrointestinal bleeding. Surg Clin North Am. (2014) 94:43–53. doi: 10.1016/j.suc.2013.10.004 24267496

[12] Garcia-Tsao G Abraldes JG Berzigotti A Bosch J. Portal hypertensive bleeding in cirrhosis: risk stratification, diagnosis, and management: 2016 practice guidance by the American association for the study of liver diseases. Hepatology. (2017) 65:310–35. doi: 10.1002/hep.28906, PMID: 27786365

[13] Nett A Binmoeller KF. Endoscopic Management of Portal Hypertension-related Bleeding. Gastrointest Endosc Clin N Am. (2019) 29:321–37. doi: 10.1016/j.giec.2018.12.006 30846156

[14] Jachs M Hartl L Simbrunner B Bauer D Paternostro R Scheiner B. The sequential application of Baveno VII criteria and VITRO score improves diagnosis of clinically significant portal hypertension. Clin Gastroenterol Hepatol. (2023) 21:1854–1863.e10. doi: 10.1016/j.cgh.2022.09.032, PMID: 36244661

[15] Ibrahim EH Marzouk SA Zeid AE Lashen SA Taher TM. Role of the von Willebrand factor and the VITRO score as predictors for variceal bleeding in patients with hepatitis C-related cirrhosis. Eur J Gastroenterol Hepatol. (2019) 31:241–7. doi: 10.1097/MEG.0000000000001272, PMID: 30281535

[16] Deng H Qi X Peng Y Li J Li H Zhang Y. Diagnostic accuracy of APRI, AAR, FIB-4, FI, and king scores for diagnosis of esophageal varices in liver cirrhosis: a retrospective study. Med Sci Monit. (2015) 21:3961–77. doi: 10.12659/MSM.895005, PMID: 26687574 PMC4690652

[17] Tarzamni MK Somi MH Farhang S Jalilvand M. Portal hemodynamics as predictors of high risk esophageal varices in cirrhotic patients. World J Gastroenterol. (2008) 14:1898–902. doi: 10.3748/wjg.14.1898, PMID: 18350629 PMC2700414

[18] Zardi EM Di Matteo FM Pacella CM Sanyal AJ. Invasive and non-invasive techniques for detecting portal hypertension and predicting variceal bleeding in cirrhosis: a review. Ann Med. (2014) 46:8–17. doi: 10.3109/07853890.2013.857831, PMID: 24328372 PMC4904298

[19] Castera L Pinzani M Bosch J. Non invasive evaluation of portal hypertension using transient elastography. J Hepatol. (2012) 56:696–703. doi: 10.1016/j.jhep.2011.07.005 21767510

[20] Liu H Sun J Liu G Liu X Zhou Q Zhou J. Establishment of a non-invasive prediction model for the risk of oesophageal variceal bleeding using radiomics based on CT. Clin Radiol. (2022) 77:368–76. doi: 10.1016/j.crad.2022.01.046, PMID: 35241274

[21] Luo R Gao J Gan W Xie WB. Clinical-radiomics nomogram for predicting esophagogastric variceal bleeding risk noninvasively in patients with cirrhosis. World J Gastroenterol. (2023) 29:1076–89. doi: 10.3748/wjg.v29.i6.1076, PMID: 36844133 PMC9950861

[22] Chinese Society of Hepatology CMA. Chinese guidelines on the management of liver cirrhosis. Zhonghua Gan Zang Bing Za Zhi. (2019) 27:846–65. doi: 10.3760/cma.j.issn.1007-3418.2019.11.008 31941240 PMC12770630

[23] Gines P Cardenas A Arroyo V Rodes J. Management of cirrhosis and ascites. N Engl J Med. (2004) 350:1646–54. doi: 10.1056/NEJMra035021 15084697

[24] Jalan R Szabo G. New concepts and perspectives in decompensated cirrhosis. J Hepatol. (2021) 75:S1–2. doi: 10.1016/j.jhep.2020.12.008 34039481 PMC9272485

[25] Steele S Lin F Le TL Medline A Higgins M Sandberg A. Segmentation and linear measurement for body composition analysis using slice-O-Matic and Horos. J Vis Exp. (2021) 169:e61674. doi: 10.3791/61674-v 33818558

[26] Zeng X Shi ZW Yu JJ Wang LF Luo YY Jin SM. Sarcopenia as a prognostic predictor of liver cirrhosis: a multicentre study in China. J Cachexia Sarcopenia Muscle. (2021) 12:1948–58. doi: 10.1002/jcsm.12797, PMID: 34520115 PMC8718091

[27] Koo TK Li MY. A guideline of selecting and reporting Intraclass correlation coefficients for reliability research. J Chiropr Med. (2016) 15:155–63. doi: 10.1016/j.jcm.2016.02.012, PMID: 27330520 PMC4913118

[28] Tibshirani R. Regression shrinkage and selection via the Lasso: a retrospective. J R Stat Soc Series B Stat Methodol. (2011) 73:273–82. doi: 10.1111/j.1467-9868.2011.00771.x

[29] Vickers AJ Elkin EB. Decision curve analysis: a novel method for evaluating prediction models. Med Decis Mak. (2006) 26:565–74. doi: 10.1177/0272989X06295361, PMID: 17099194 PMC2577036

[30] Topan MM Sporea I Danila M Popescu A Ghiuchici AM Lupusoru R. Impact of sarcopenia on survival and clinical outcomes in patients with liver cirrhosis. Front Nutr. (2021) 8:766451. doi: 10.3389/fnut.2021.766451, PMID: 34746216 PMC8566695

[31] Montano-Loza AJ Duarte-Rojo A Meza-Junco J Baracos VE Sawyer MB Pang JX. Inclusion of sarcopenia within MELD (MELD-sarcopenia) and the prediction of mortality in patients with cirrhosis. Clin Transl Gastroenterol. (2015) 6:e102. doi: 10.1038/ctg.2015.31 26181291 PMC4816259

[32] Ebadi M Tsien C Bhanji RA Dunichand-Hoedl AR Rider E Motamedrad M. Myosteatosis in cirrhosis: a review of diagnosis, pathophysiological mechanisms and potential interventions. Cells. (2022) 11:1216. doi: 10.3390/cells11071216, PMID: 35406780 PMC8997850

[33] Sharma P. Value of liver function tests in cirrhosis. J Clin Exp Hepatol. (2022) 12:948–64. doi: 10.1016/j.jceh.2021.11.004, PMID: 35677506 PMC9168739

[34] Bernardi M Maggioli C Zaccherini G. Human albumin in the management of complications of liver cirrhosis. Crit Care. (2012) 16:211. doi: 10.1186/cc11218, PMID: 22429536 PMC3681351

[35] Li J Qi X Deng H Peng Y Shao L Ma J. Association of conventional haemostasis and coagulation tests with the risk of acute upper gastrointestinal bleeding in liver cirrhosis: a retrospective study. Gastroenterol Rep (Oxf). (2016) 4:315–9. doi: 10.1093/gastro/gov059, PMID: 26672007 PMC5193061

[36] Mayerhoefer ME Materka A Langs G Haggstrom I Szczypinski P Gibbs P. Introduction to Radiomics. J Nucl Med. (2020) 61:488–95. doi: 10.2967/jnumed.118.222893, PMID: 32060219 PMC9374044

[37] Chen XD Chen WJ Huang ZX Xu LB Zhang HH Shi MM. Establish a new diagnosis of sarcopenia based on extracted Radiomic features to predict prognosis of patients with gastric Cancer. Front Nutr. (2022) 9:850929. doi: 10.3389/fnut.2022.850929, PMID: 35845809 PMC9276522

